# VEGF (Vascular Endothelial Growth Factor) and Fibrotic Lung Disease

**DOI:** 10.3390/ijms19051269

**Published:** 2018-04-24

**Authors:** Shaney L. Barratt, Victoria A. Flower, John D. Pauling, Ann B. Millar

**Affiliations:** 1 Academic Respiratory Unit, School of Clinical Sciences, University of Bristol, Bristol BS10 5NB, UK; ann.millar@bristol.ac.uk; 2 Department of Pharmacy and Pharmacology, University of Bath, Bath BA1 1RL, UK; V.A.Flower@bath.ac.uk (V.A.F.); j.d.pauling@bath.ac.uk (J.D.P.)

**Keywords:** interstitial lung disease, fibrosis, Vascular Endothelial Growth Factor, VEGF

## Abstract

Interstitial lung disease (ILD) encompasses a group of heterogeneous diseases characterised by varying degrees of aberrant inflammation and fibrosis of the lung parenchyma. This may occur in isolation, such as in idiopathic pulmonary fibrosis (IPF) or as part of a wider disease process affecting multiple organs, such as in systemic sclerosis. Anti-Vascular Endothelial Growth Factor (anti-VEGF) therapy is one component of an existing broad-spectrum therapeutic option in IPF (nintedanib) and may become part of the emerging therapeutic strategy for other ILDs in the future. This article describes our current understanding of VEGF biology in normal lung homeostasis and how changes in its bioavailability may contribute the pathogenesis of ILD. The complexity of VEGF biology is particularly highlighted with an emphasis on the potential non-vascular, non-angiogenic roles for VEGF in the lung, in both health and disease.

## 1. Introduction

The term ‘fibrotic lung disease’ or interstitial lung disease (ILD) encompasses a group of more than 100 heterogeneous diseases characterized by similar clinical and radio-pathological patterns of aberrant inflammation and fibrosis of the lung parenchyma despite a wide variety of potential triggers and prognoses [1]. Accurate diagnosis depends on thorough assessment of potential contributing aetiologies, including drugs, granulomatous disease, occupational or environmental exposures (Hypersensitivity pneumonitis–HP) and connective tissue disorders (CTD), but may occur secondary to an unknown cause and are termed the Idiopathic ILDs. Some of these are potentially reversible, such as acute respiratory distress syndrome (ARDS) whilst others are inexorably progressive such as Idiopathic Pulmonary Fibrosis (IPF).

Whilst the exact pathogenesis of each disease may differ, they are characterised by a pathologic fibrotic-repair mechanism following epithelial and endothelial cell injury with aberrant vascular remodelling, expansion and activation of the lung fibroblast/myofibroblast population with resulting abnormal accumulation of extracellular matrix (ECM) and architectural distortion.

Over the last decade there has been growing interest in the role of Vascular Endothelial Growth Factor (VEGF) in the pathogenesis of ILD, with the development of nintedanib for the treatment of IPF, a novel triple tyrosine kinase inhibitor of VEGF, fibroblast derived growth factor (FGF) and platelet derived growth factor (PDGF) receptors [2].

This review describes our current understanding of VEGF biology, highlighting its potential role in normal lung homeostasis and in ILD pathogenesis, with a particular focus in ARDS, IPF, HP and CTD-ILD.

A detailed account of recent advances in VEGF signaling is beyond the scope of this review and is provided elsewhere within this themed collection. Nonetheless, we shall briefly report VEGF biology with respect to its relationship with lung homeostasis and disease. Whilst not systematic in nature, we shall draw on a number of sources, including preclinical mechanistic studies, clinical research and clinical trial data.

## 2. VEGF Biology

### 2.1. VEGF Isoforms

VEGF-A is a 34–46 kDa glycoprotein that belongs to a superfamily of structurally and functionally related proteins that includes VEGF-B, VEGF-C, VEGF-D, VEGF-E and placental growth factor (PlGF) [3]. Whilst VEGF-A was originally described as a key regulator of angiogenesis [3,4], there has been significant evolution of the understanding of VEGF biology over the last three decades such that the initial description can be considered as a misnomer; VEGF has been identified in nematode species who lack any vasculature [5], and expression or targeted function is not specific to endothelial cells [6,7].

**Figure 1:**
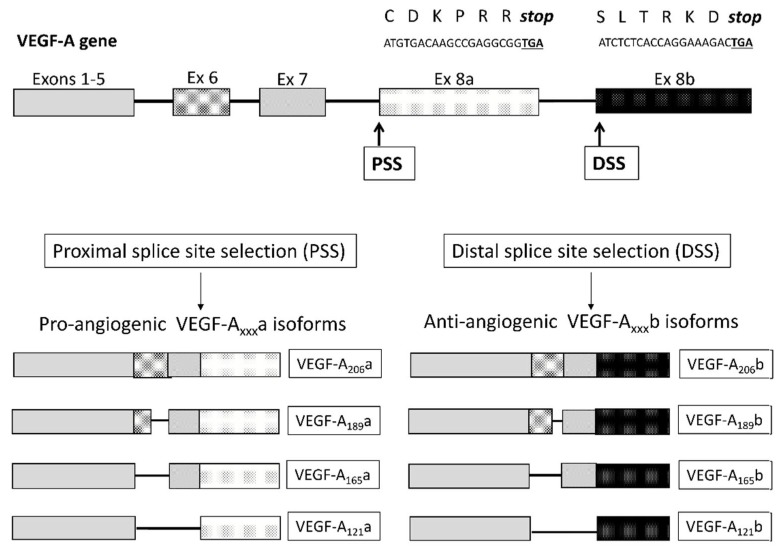
Schematic diagram of the exonic structure of the *Vascular Endothelial Growth Factor-A* (*VEGF-A*) gene and its splice isoforms. The *VEGF-A* gene consists of 8 exons separated by 7 introns. Two alternative exon 8 splice sites exist. Differential splicing of VEGF-A mRNA from exons 5 to 8, with proximal splice site (PSS) selection in exon 8 (Ex8a) generates human isoforms, collectively termed the VEGF-A_xxx_a isoforms: including VEGF-A_121_a, VEGF-A_165_a, VEGF-A_189_a and VEGF-A_206_a, where the subscript denotes the number of amino acids. Distal splice site selection (DSS) produces a second family of isoforms, the VEGF-A_xxx_b proteins which have the same number of amino acids as the conventional VEGF-A_xxx_a isoforms but have an alternative amino acid sequence at their carboxy-terminal (C-terminal) domain: Ser-Leu-Thr-Arg-Lys-Asp (SLTRKD) instead of Cys-Asp-Lys-Pro-Arg-Arg (CDKPRR) in VEGF-A_xxx_a isoforms. TGA represents the stop codon (***stop***).

The *VEGF-A* gene consists of 8 exons separated by 7 introns. Differential splicing of VEGF-A mRNA from exons 5 to 8 generates six known human isoforms, collectively termed the VEGF-A_xxx_a isoforms: VEGF-A_121_a, VEGF-A_145_a, VEGF-A_165_a, VEGFA_183_a, VEGF-A_189_a and VEGF-A_206_a, where the subscript denotes the number of amino acids (Figure 1) [3]. VEGF-A_165_a is considered to be most abundant of these isoforms, functioning through tyrosine kinase receptors VEGF receptor 1 (VEGFR1) and receptor 2 (VEGFR2) and co-receptors Neuropilin 1 (NP1) and (NP2).

Differential splicing of the VEGF gene at the distal splice site with exon 8; 66 bp distal to the VEGF-A_xxx_a acceptor-site, produces a second family of isoforms, the VEGF-A_xxx_b proteins which have the same number of amino acids as the conventional VEGF-A_xxx_a isoforms but have an alternative amino acid sequence at their carboxy-terminal (C-terminal) domain: Ser-Leu-Thr-Arg-Lys-Asp (SLTRKD) instead of Cys-Asp-Lys-Pro-Arg-Arg (CDKPRR) in VEGF-A_xxx_a isoforms (Figure 1) [8]. The most widely studied of these isoforms, VEGF-A_165_b, has been shown to act as an inhibitor of VEGF-A165a [8,9] through competitive interference with the VEGFR2-NP1 complex and activation of different downstream receptor phosphorylation sites [10]. Because of sequence homology between these isoform families, a precise, isoform-specific methodology is required to differentiate between them [11].

VEGF-A is the most widely studied molecule of the VEGF superfamily, but it may form heterodimer complexes with other family members to activate VEGF receptors [12] and modulate downstream signalling [13]. VEGF-B is particularly abundant in the heart and skeletal muscle [14] and may contribute to the pulmonary vascular remodelling occurring in response to chronic hypoxia exposure [15]. VEGF-C and VEGF-D are key mediators of lymphangiogenesis [16,17]. VEGF-E is an Orf virus-encoded VEGF homologue which although not present in the human genome binds specifically to VEGFR2 [18]. In normal tissues, PlGF is present most abundantly in the placenta, thyroid and lungs, although its exact role in these tissues remains unclear [19]. When PlGF is produced in the same population of cells with VEGF, it can act as a natural occurring competitive inhibitor [20,21].

### 2.2. VEGF Receptors

VEGFR2 (also known as kinase domain region (KDR) or fetal liver kinase-1 (FLK-1)) is considered by many as the main signalling receptor for VEGF bioactivity [22,23]. It is abundantly expressed in the vascular bed where it appears to be critical for normal development [24], but several non-endothelial cells (non-ECs), including lung macrophages [3] and alveolar epithelial type II (ATII) cells [25] have also been shown to express VEGFR2.

VEGFR1 (or Flt-1 (Fms-like tyrosine kinase 1) in the mouse) is a 180–185 kDa glycoprotein [26], which also exists as an alternatively spliced soluble isoform (sFlt). Like VEGFR2, VEGFR1 is expressed in high levels throughout development and in adulthood within the vascular bed and is also expressed by several non-ECs, including in lung macrophages, monocytes [27] and ATII cells [28,29]. The exact roles of both VEGFR1 and sFlt are not fully understood, although an abundance of evidence indicates that they function as ‘decoy’ receptors, sequestering VEGF, thus limiting its availability to bind to VEGFR2 [30,31]. Several studies dispute this however, directly implicating it in the regulation of EC migration [32] and survival [33].

NP1 and NP2 are transmembrane glycoproteins, which notably have a short cytoplasmic domain and as such are thought to transduce functional responses only when co-expressed with other receptors [34,35]. Contrasting evidence exists, however, suggesting that NP1 is able to support VEGF-induced cellular signalling independent of VEGFR2 [36] and may have an independent role in the maintenance of normal lung structure [37].

### 2.3. VEGF and the Lung

In utero, the alveoli, airways and pulmonary vasculature all develop in synchrony [38]. Airway epithelial cells are the predominant source of VEGF-A throughout lung organogenesis [39] and it appears to be crucial for normal alveolarisation, rapid alveolar multiplication during lung maturation [40,41] and normal development of the vascular bed [42]. VEGF-B, VEGF-C, VEGF-D and PlGF are also thought to play a role in physiological lung development but have not been widely studied [43,44,45,46].

Following birth, the human lung continues to undergo a period of maturation with rapid alveolar multiplication up to the age of 2 years [47]. Several animal studies also implicate VEGF-A as a crucial factor in this process [42,48,49].

Significant amounts of VEGF-A persist in the normal adult lung, where again the alveolar epithelium [28,50,51,52] appears to be the prominent source, although smooth muscle cells, macrophages, ECs and fibroblasts [52] also express VEGF-A [53,54]. Recently it has been shown that this VEGF-A represents both VEGF-A_xxx_a and VEGF-A_xxx_b isoforms [52]. Likewise, VEGF receptors and co-receptors are also expressed by several cell types within the normal lung, on both sides of the alveolar capillary membrane (ACM) including ATII cells [25,28,29,55], ECs [24,56,57], macrophages [3,27] and fibroblasts [52].

The classical processes linked to VEGF-A activity (permeability, angiogenesis and mitogenesis) are extremely limited in the mature lung. Thus, whilst the exact role of VEGF-A in the lung has not been fully defined, it has been proposed that compartmentalisation of VEGF-A within the alveolar space, by way of an intact ACM [51,58], is imperative for maintaining normal lung structure and function. ACM disruption is considered part of the disease pathogenesis of ARDS, IPF, HP and systemic sclerosis (SSc), albeit by potentially differing mechanisms, which supports this theory. Each mechanism will be discussed in more detail in the relevant sections.

VEGF-A has been observed to stimulate ATII growth [59,60], surfactant production [61] and angiogenesis of the systemic vasculature [4] with reports suggesting an additional anti-apoptotic and survival role for epithelial [62,63,64] and ECs [65,66,67], as such a role for VEGF-A in lung repair following injury has been proposed. Both VEGF-A blockade [42,68,69,70] and VEGF-A overexpression [71] have been reported to result in an emphysema phenotype in pre-clinical models, suggesting tight regulation of VEGF-A expression as part of lung homeostasis, whilst others have observed the development of pulmonary oedema secondary to VEGF-A overexpression [72]. The role of other VEGF family members in lung homeostasis is not well defined, although PlGF overexpression in pre-clinical animal models also appears to induce emphysematous change [73].

## 3. VEGF in ARDS

ARDS is a form of diffuse lung injury characterised by the onset of refractory hypoxaemia associated with bilateral lung infiltrates that are not associated with cardiac failure or fluid overload and occur following trigger insult [74]. Damage to the ACM is central to disease pathogenesis, with resulting increased vascular permeability and accompanied inflammatory cell migration and proteinaceous fluid exudation into the lung parenchyma (exudative phase) [75]. Recovery from ARDS is thought to require repair of the ACM through a co-ordinated process of ATII cell proliferation with resorption of the oedema and clearance of proteinaceous material. Whilst the changes associated with ARDS may fully resolve, a proportion of patients heal by fibrin deposition and the development of pulmonary fibrosis (fibroproliferative response) [76], but the factors determining this are not completely understood. 

As a potent angiogenic and permeability factor, which is thought to be critical for the structure and maintenance of the normal lung, VEGF-A has been proposed as a key factor in the pathogenesis of this disease [58,76]. VEGF polymorphisms have been associated with both increased severity of and mortality from ARDS [77,78,79], suggesting that genetic factors may have a role.

Several studies support that VEGF-A contributes as a protective factor against ARDS [80], with observations of reduced bronchoalveolar lavage fluid (BALF) and increased plasma VEGF-A in early ARDS and normalisation in recovery [81,82,83]. Expression of ATII-derived VEGF-A is increased during recovery from experimental lung injury, implicating VEGF-A in the ACM repair process [84]. Furthermore, the overexpression of VEGF-A_165_a in distal lung epithelial cells confers cytoprotection against experimental hyperoxic lung injury, in part mediated through the production of anti-apoptotic proteins [85]. 

In contrast, others have suggested a pathological role for VEGF-A in ARDS with the development of pulmonary oedema and increased capillary permeability following adenoviral delivery of VEGF-A_165_a into the trachea of mice, an effect mitigated by anti-VEGF-A therapy [86,87]. Whilst methodological diversity might explain these apparently contrasting findings, we also proposed that the identification of VEGF-A_xxx_a and VEGF-A_xxx_b isoforms, with seemingly opposing effects both in vitro and in vivo, may also provide an alternative explanation [78]. In vitro, VEGF-A_165_b was found to inhibit the proliferative effect of VEGF-A_165_a on human primary ECs and ATII cells, with reduced expression of VEGF-A_165_b in ARDS compared to the normal lung, suggesting a role for VEGF-A_xxx_b in the repair of the ACM following lung injury [60].

The contribution of VEGF-A to the fibro-proliferative phase of ARDS has not been specifically addressed, as far as the authors are aware, although several studies have established a role for VEGF-A in the development of IPF, and these are discussed separately in this article.

A planned phase 2 clinical trial studying the efficacy of the anti-VEGF monoclonal antibody bevacizumab in preventing ARDS (NCT01314066) was recently withdrawn, prior to enrolment, due to inadequate funding. As such, there are no disease modifying therapies currently available for ARDS and supportive care, and lung protective ventilator strategies remain the mainstay of treatment.

## 4. VEGF in IPF

IPF is the most common of the idiopathic ILDs associated with high mortality; estimated as greater than 50 per 1,000,000 persons [88,89] and an estimated mean survival of only 2–5 years from diagnosis [90,91]. Best supportive care for these patients includes consideration of pharmacological options such as pirfenidone [92] and the triple tyrosine kinase inhibitor (VEGF, FGF and PDGF) nintedanib [2], which attempt to slow disease progression with both pharmacological and non-pharmacological interventions to palliate symptoms. 

The pathogenesis of IPF remains poorly understood, although alveolar epithelial cell injury [1,93] with disruption of ACM integrity alongside abnormal vascular repair and remodelling, have been proposed as possible pathogenic mechanisms [94,95,96]. Ultimately, the formation of collections of fibroblasts and activated myofibroblasts (fibroblastic foci) appear to be at the leading edge of this disease [97], producing the exaggerated extracellular matrix (ECM) deposit that contributes to the disruption of normal lung architecture.

The relationship of VEGF-A expression in IPF remains controversial and appears to differ according to the compartment sampled. Several groups have observed reduced VEGF-A in the BALF of IPF patients compared to controls [52,94,98,99,100], whilst others have reported unchanged levels [101]. Similarly, VEGF-A in lung homogenates are reduced [101] or unchanged [52,94] in IPF. Equally, there are contrasting reports as to the trend of circulating VEGF-A levels in IPF patients relative to the severity and progression of the disease [52,99,101,102].

As a potent angiogenic factor, interest arose into whether VEGF-A may contribute to the vascular remodelling process [95,103]. Minimal VEGF-A expression has been demonstrated within the fibrotic focus itself [52,94], but is expressed in abundance in the surrounding tissue [52]. Increased alveolar capillary density in non-fibrotic regions of the IPF lung has also been associated with the expression of VEGF-A and other potent angiogenic mediators by ATII cells in close proximity to these capillaries [95]. The primary vascular abnormality in IPF, be it a lack or excess of neovascularisation is still unknown, and equally, the role of increased vascularisation in the least fibrotic regions has not been defined [38,104,105]. Given that VEGF-A potentially plays a role in normal lung maintenance and repair, it has been hypothesised that in relatively normal areas of the IPF lung, VEGF-A released from ATII cells may play a role in alveolar wall protection, contributing to the regeneration of wall defects; with locally increased vascularity occurring as part of the attempted repair process [105]. Several studies support this hypothesis, suggesting a protective role for VEGF-A against the formation of pulmonary fibrosis [101,106,107] and Murray et al. [101] have recently proposed that this epithelial-protective function of VEGF-A may occur via a non-cell autonomous function mediated by the endothelium.

Fehrenbach et al. [25] were amongst the first groups to suggest that VEGF–A may have a wider part to play in the development of pulmonary fibrosis, rather than only on the vasculature, by demonstrating a marked increase in VEGF-A positive stained cells in the absence of increased vascularisation in the fibrotic regions in a preclinical model of pulmonary fibrosis (Bleomycin (BLM)-induced pulmonary fibrosis). Subsequently, Hamada et al. [108] proposed that VEGF-A might facilitate fibrogenesis. Transfection of anti-VEGF gene therapy, in the form of the sFlt-1, resulted in the attenuation of pulmonary fibrosis with a reduction in lung collagen deposition and additional anti-inflammatory and anti-angiogenic effects. Furthermore, Chaudhary et al. [109] demonstrated that BIBF 1000, a novel tyrosine kinase inhibitor of PDGF, FGF and VEGF, attenuated BLM-induced pulmonary fibrosis in rats, as measured by a reduction in collagen deposition and the inhibition of pro-fibrotic gene expression. This compound is now available clinically as Nintedanib and has been approved for the treatment of IPF based on the results of twin Phase III INPULSIS-1 and -2 trials [2].

Therefore, as was the case for ARDS, results from the currently available evidence suggests potentially conflicting roles for VEGF-A as both a protective and contributory factor in the development of IPF. Interestingly, in pre-clinical studies, the concomitant adenoviral delivery of TGF-β1 and VEGF-A_165_a results in exaggerated pulmonary fibrosis, but attenuation of pulmonary artery remodelling and pulmonary hypertension, compared to TGF-β1 alone [110], highlighting the complicated role that VEGF-A may play in the lung, with potentially opposing effects of VEGF-A in different lung compartments existing concurrently.

An alternative explanation for the apparently contradicting data regarding the role of VEGF-A in IPF has recently been proposed. The co-ordinated expression of VEGF-A_xxx_a and VEGF-A_xxx_b isoforms are important for the development of pulmonary fibrosis both in vitro and in pre-clinical murine models [52]. In this study, ATII cell-derived VEGF-A_xxx_a was critical for the development of fibrosis in a preclinical model of fibrosis, with an inhibitory/regulatory function for VEGF-A_xxx_b isoforms. Furthermore, VEGF_165_a and VEGF-A_165_b had differential effects on fibroblast proliferation, migration and ECM production in vitro. Up-regulation of VEGF-A_165_b within the IPF lung and in patients who progressed after 1 year follow-up (Forced Vital Capacity (FVC) fall of ≥10% or death), suggests that the VEGF-A_xxx_b may be released as a compensatory protective mechanism against fibrogenesis, overwhelmed by other processes occurring within the lung. 

## 5. VEGF in Hypersensitivity Pneumonitis (HP)

HP is an interstitial lung disease characterised by inflammation and/or fibrosis in susceptible individuals following repeated inhalation of environmental antigens. As only a small proportion of individuals exposed to a particular antigen develop the disease, paradigms suggest a two-hit hypothesis with an additional genetic predisposition [111]. A clinical spectrum of disease exists with acute presentations thought to be mediated through immune complexes, as suggested by lung neutrophilia and high titres of antigen-specific serum IgGs, whilst sub-acute and chronic presentations are characterised by a T-cell-mediated immune response [112].

Progressive fibrosis may ensue, if responsible antigens are not identified and continued exposure occurs, with associated excessive extracellular matrix deposition and the destruction of normal lung architecture. The processes driving this are less well understood, although differences in gene expression profiling [113], BALF cellular content and cytokine expression between IPF and HP suggests mechanistic divergence in the pathogenesis of fibrosis between these two conditions [114,115]. That said, the upregulation of the markers of alveolar epithelial apoptosis in human lung sections from patients with HP [116] suggests that alveolar epithelial cell integrity is again important in the disease process.

Very few studies have examined a potential role for VEGF-A in HP. In the small cohorts examined thus far, analogous to ARDS and IPF, BALF VEGF-A levels are reduced in patients with HP [115,117]. In contrast, serum VEGF-A levels appear increased compared to controls [117,118] and IPF patients [118].

The function of the lymphatic system is primarily to transport antigens and antigen-presenting cells from the peripheral tissues to lymph nodes to stimulate an immune response [119]. Lymphangiogenesis occurs in various pathological conditions, including during inflammation and wound healing. As key mediators of lymphangiogenesis, a role for VEGF-C and VEGF-D in the development of HP has thus been proposed. In a small cohort of acute and subacute HP patients, BALF VEGF-C and VEGF-D levels were elevated compared to healthy controls, with increased levels of VEGF-D but not VEGF-C compared to IPF patients. Furthermore, VEGF-D levels correlated with HP inflammatory severity as determined by BALF lymphocytosis [118]. Further work is required to explore this apparent association.

## 6. VEGF-A in Autoimmune Rheumatic Diseases

Dysregulated tissue remodeling with aberrant fibrosis is one of the pathological hallmarks of the autoimmune rheumatic diseases and ILD is an important cause of disease-related morbidity across this group of disorders, particularly within connective tissue diseases (CTD) such as SSc [120].

### 6.1. SSc

SSc is a multisystem disease characterised by a triad of autoimmunity, vasculopathy and aberrant tissue remodeling resulting in varying degrees of tissue fibrosis [121,122]. SSc-ILD is the leading cause of disease-related mortality [123]. Endothelial injury is an important initiating pathological event [124,125,126] and clinical manifestations of vasculopathy (characteristic nailfold capillary changes and Raynaud’s phenomenon) pre-date the development of tissue fibrosis [127]. The evolving obliterative microangiopathy characterized by progressive capillary loss (that can be directly visualized at the nailfold) results in progressive tissue ischaemia, which could be an important driver of both ischaemic complications such as digital ulcers but also tissue fibrosis [128,129].

The induction of VEGF pathways by hypoxia [130] has led to interest in its potential role in the pathogenesis of SSc. Early studies demonstrated raised circulating levels of VEGF-A in both early [131] and more established SSc [132]; surprising given the progressive capillary loss in SSc. Subsequent work examining VEGF-A splice isoforms provided a plausible explanation, having identified increased plasma levels of the VEGF-A_165_b splice variant in association with more severe nailfold capillary loss [133]. It is possible that isoform switching from pro-angiogenic VEGF-A_xxx_a isoform production in early disease to inhibitory VEGF-A_xxx_b isoforms might help explain disease evolution in this heterogeneous disease, although the mechanisms leading to isoform switching have yet to be elucidated. With regards to SSc-ILD, there are lower VEGF-A BALF levels in SSc compared to both healthy controls and SSc patients without lung involvement [134]. De Santis et al. observed a direct correlation between circulating VEGF-A and increased severity of ILD, as determined by the extent of interstitial abnormalities on CT imaging and lung function parameters, suggesting a possible pathological role for VEGF-A in SSc-ILD [135]. The anti-angiogenic VEGF-A_165_b isoform has yet to be fully investigated in SSc-related pulmonary disease. Nintedanib has recently been shown to ameliorate histological features of pulmonary arterial hypertension (PAH) and pulmonary fibrosis in pre-clinical models of SSc, which has encouraging implications for ongoing phase III clinical trials of nintedanib in SSc-associated ILD [136].

### 6.2. Other Forms of CTD-ILD

Both VEGF-A and anti-angiogenic VEGF-A_165_b isoforms are over-expressed in muscle tissue from patients with myositis-spectrum disorders (MSD) compared to healthy donors [137,138]. However, there is limited data on circulating VEGF-A levels and pulmonary disease in MSD [139]. The only work examining VEGF in systemic lupus erythematosus (SLE)-related lung disease has focused on PAH, identifying higher levels of VEGF-A in SLE patients with PAH compared to those without [140]. Similar results were found in PAH related to mixed connective tissue disease [141]. Microscopic polyangiitis (MPA) is a systemic small vessel vasculitis with pulmonary involvement ranging from ILD, nodularity, consolidation and pleural effusions. Serum VEGF-A is increased in MPA patients (with lung involvement) and falls in response to systemic immunosuppression, perhaps because inflammatory cells such as macrophages are an important source [142].

### 6.3. Inflammatory Arthritis

Rheumatoid arthritis (RA) is common (prevalence ~1%), but clinically meaningful RA-associated ILD is rare. Circulating VEGF-A is increased in RA patients, particularly in those patients with extra-articular manifestations (including pulmonary fibrosis) [143,144].

## 7. Summary

Significant quantities of VEGF-A exist in the normal lung. Processes classically associated with VEGF-A (angiogenesis, mitogenesis and permeability) are extremely restricted, however, suggesting an alternative role for VEGF-A in the mature lung. Growing evidence suggests that this role involves the maintenance of normal lung structure and function, where an intact ACM and thus compartmentalisation of VEGF-A appears crucial.

**Figure 2:**
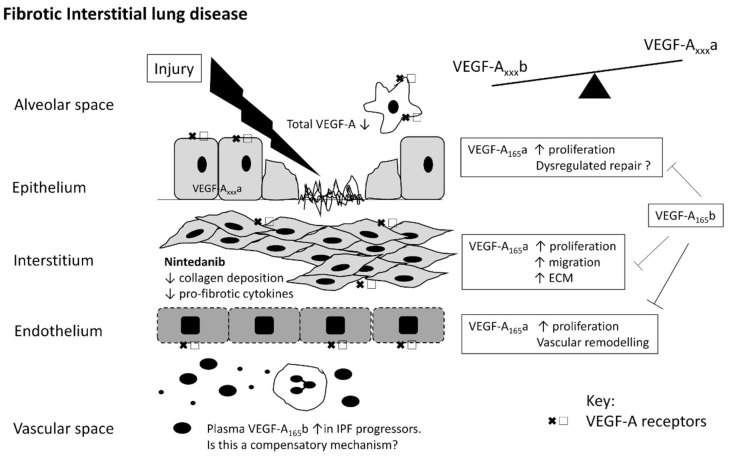
Schematic diagram of the role of VEGF-A in the development of fibrotic interstitial lung disease. Current paradigms suggest repeated alveolar epithelial cell injury is an important initiating factor. VEGF-A receptors are abundantly expressed on both sides of the alveolar capillary membrane; alveolar epithelial type II (ATII) cells [25,28,29,55], macrophages [3,27], in the vascular bed [24,56,57] and by fibroblasts [52]. Total VEGF-A levels are consistently reduced in the bronchoalveolar lavage fluid of patients with fibrotic lung disease. Nintedanib is a tyrosine kinase inhibitor of VEGF-A receptor activity [2] (thus theoretically inhibiting VEGF-A_xxx_a and VEGF-A_xxx_b isoforms) with clinical application in the treatment of idiopathic pulmonary fibrosis (IPF) [2]. ATII cell derived VEGF-A_xxx_a appears critical for the development of pulmonary fibrosis in pre-clinical models, with VEGF-A_165_b having an inhibitory/opposing effect [52]. In vitro, VEGF-A_165_a has been shown to induce the proliferation of ATII cells [60], endothelial cells [60] and fibroblasts [52], and increase extracellular matrix production by fibroblasts [52], all inhibited by VEGF-A_165_b. Taken in conjunction with the data from pre-clinical models it suggests that the co-ordinated expression of VEGF-A_xxx_a:VEGF-A_xxx_b appears important in health and disease, with VEGF-A_xxx_a acting as a driver of the fibrotic process. Upregulation of circulating VEGF-A_165_b levels in IPF patients who subsequently progressed after 1 year follow-up (FVC fall of ≥10% or death), suggests that VEGF-A_xxx_b may be released as a compensatory protective mechanism against fibrogenesis, overwhelmed by other processes occurring within the lung [52].

There are apparent disparities in the literature regarding VEGF-A in lung disease, which may be in part due to methodological differences in the study design and animal models used. It is possible that regional or compartmental differences in VEGF-A expression in the lung or heterogeneity within and between the individuals studied may also account for the differences observed. The presence of and differential influence of VEGF-A splice variants offers an alternative explanation (Figure 2).

The complexity of VEGF biology in lung disease is becoming increasingly apparent, not to mention the numerous physiological roles of VEGF in several organ systems and the potential for pleiotropic effects [145,146]. The development of future therapies directed at VEGF requires consideration of these factors with detailed characterisation of patient phenotypes to enable superior targeted therapy.
